# Supplementary material for: Repurposing of Miltefosine as an Adjuvant for Influenza Vaccine
Source: Vaccines (Basel). 2020 Dec 11;8(4):754. doi: 10.3390/vaccines8040754 (PMC7768360; doi:10.3390/vaccines8040754)
Supplement: Supplementary file 1 [file vaccines-08-00754-s001.pdf]

Supplementary Table S1 Vaccines used in this project

Vaccine	Usage	Season	Lot No.	Content
**Fluarix® Tetravalent**	Vaccination-challenge model (7 days apart)	2014-15	AFLBA032AA	A/California/7/2009(H1N1)pdm09-like strain
				A/Texas/50/2012(H3N2)-like strain
				B/Massachusetts/02/2012-like strain
				B/Brisbane/60/2008-like strain
**VAXIGRIP® Trivalent**	Vaccination-challenge model (7 days apart) and mechanistic study	2015	M7080-2	A/California/7/2009(H1N1)pdm09-derived strain
				A/Switzerland/9715293/2013(H3N2)-like strain
				B/Phuket/3073/2013
**Fluarix® Trivalent**	Vaccination-challenge model (3 days apart)	2014-15	AFLUA864AH	A/California/7/2009(H1N1)pdm09-like strain
				A/Texas/50/2012(H3N2)-like strain
				B/Massachusetts/02/2012-like strain
**Fluarix® Tetravalent**	Vaccination-challenge model (3 days apart)	2015-16	AFLBA112AF	A/California/7/2009(H1N1)pdm09-like strain
				A/Switzerland/9715293/2013(H3N2)-like strain
				B/Phuket/3073/2013
				B/Brisbane/60/2008-like strain

Supplementary Table S2 Primers used for this project (mouse-specific)

Gene	Forward primer (5' to 3')	Reverse primer (5' to 3')
β-actin	ACGGCCAGGTCATCACTATTG	CAAGAAGGAAGGCTGGAAAAG
IFN-γ	AAGCGTCATTGAATCACACC	CGAATCAGCAGCGACTCCTT
IL-6	TGGAGTCACAGAAGGAGTGGCTAAG	TCTGACCACAGTGAGGAATGTCCAC

Supplementary Table S3 Antibodies, isotype controls, and streptavidin conjugate used for flow cytometry

Antigen (mouse) / isotype control	Fluorochrome	Clone	Purchased from
CD3ε	FITC	145-2C11	BioLegend
Armenian Hamster IgG Isotype control	FITC	HTK888	BioLegend
CD4	Alexa Fluor 700	RM4-5	BioLegend
Rat IgG2a, κ Isotype control	Alexa Fluor 700	RTK2758	BioLegend
CXCR5	Biotin	2G8	BD Biosciences
Rat IgG2a, κ Isotype control	Biotin	R35-95	BD Biosciences
Streptavidin (For detection of CXCR5)	APC	N/A	BioLegend
CD279 (PD-1)	PE	J43	BD Biosciences
Hamster IgG2, κ Isotype control	PE	B81-3	BD Biosciences
B220 (Used for compensation of PE-CD279)	PE	RA3-6B2	BioLegend

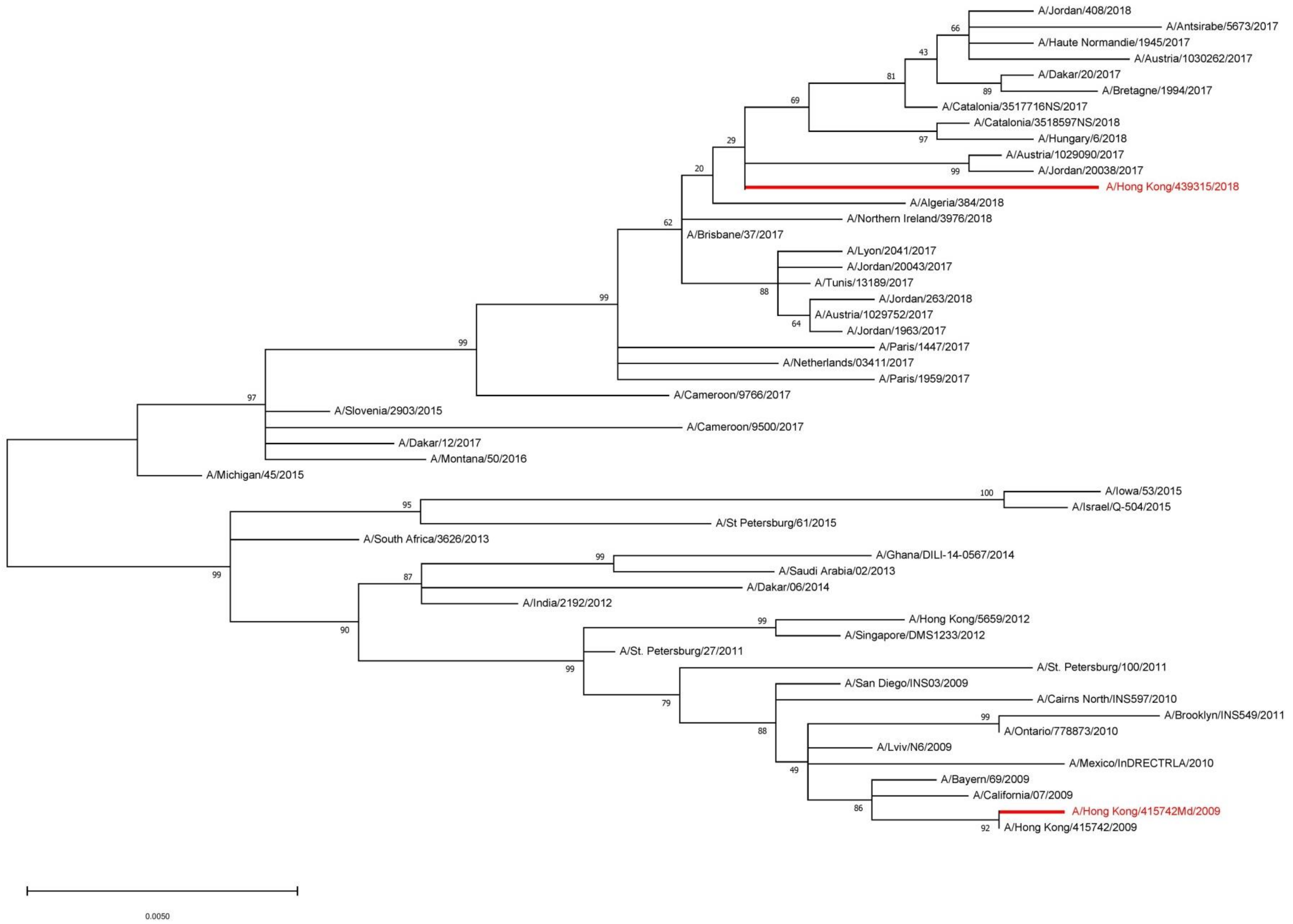
A/Jordan/408/2018
A/Antsirabe/5673/2017
A/Haute Normandie/1945/2017
A/Austria/1030262/2017
A/Dakar/20/2017
A/Bretagne/1994/2017
A/Catalonia/3517716NS/2017
A/Catalonia/3518597NS/2018
A/Hungary/6/2018
A/Austria/1029090/2017
A/Jordan/20038/2017
A/Hong Kong/439315/2018
A/Algeria/384/2018
A/Northern Ireland/3976/2018
A/Brisbane/37/2017
A/Lyon/2041/2017
A/Jordan/20043/2017
A/Tunis/13189/2017
A/Jordan/263/2018
A/Austria/1029752/2017
A/Jordan/1963/2017
A/Paris/1447/2017
A/Netherlands/03411/2017
A/Paris/1959/2017
A/Cameroon/9766/2017
A/Slovenia/2903/2015
A/Cameroon/9500/2017
A/Dakar/12/2017
A/Montana/50/2016
A/Michigan/45/2015
A/Iowa/53/2015
A/Israel/Q-504/2015
A/St Petersburg/61/2015
A/South Africa/3626/2013
A/Ghana/DILI-14-0567/2014
A/Saudi Arabia/02/2013
A/Dakar/06/2014
A/India/2192/2012
A/Hong Kong/5659/2012
A/Singapore/DMS1233/2012
A/St. Petersburg/27/2011
A/St. Petersburg/100/2011
A/San Diego/INS03/2009
A/Cairns North/INS597/2010
A/Brooklyn/INS549/2011
A/Ontario/778873/2010
A/Lviv/N6/2009
A/Mexico/InDRECTRLA/2010
A/Bayern/69/2009
A/California/07/2009
A/Hong Kong/415742Md/2009
A/Hong Kong/415742/2009
0.0050

Supplementary Figure S1 Phylogenetic tree of HA showing the genetic relationship of influenza A(H1N1) viruses. The two strains involved in the HAI and MN tests for the antibody responses against antigenically-drifted viruses are highlighted in red. The phylogenetic tree was constructed using the maximum-likelihood method with the Tamura-Nei model. Bootstrap values were calculated from 1000 trees. Evolutionary analyses were conducted in MEGA X [1].

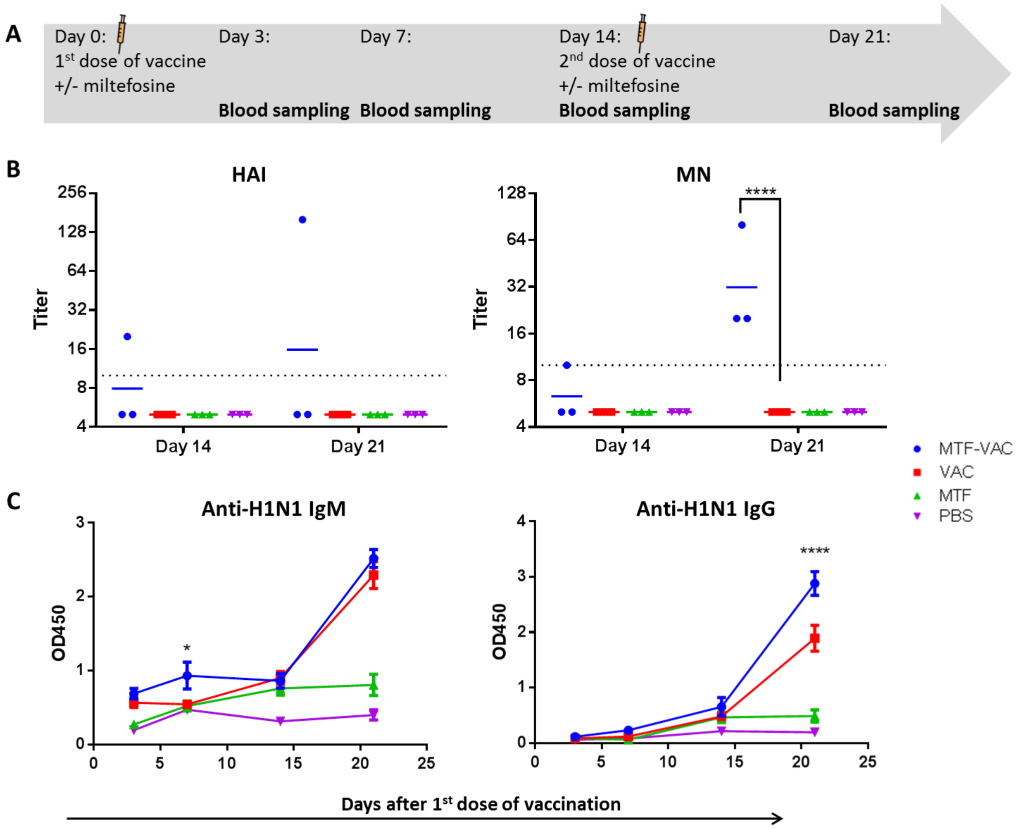

Supplementary Figure S2 The effect of two doses of vaccines with or without miltefosine on the antibody titers without influenza virus challenge. (A) Schedule of influenza vaccination and sample collection. (B) HAI titers and MN titers of serum samples after vaccination. (C) Anti-H1N1 IgM and anti-H1N1 IgG antibody levels determined by ELISA.

MTF-VAC, miltefosine (0.2 mg) + vaccine group; Vac, vaccine only group; MTF, miltefosine (0.2 mg) only group; PBS, PBS only group. Short solid lines indicate geometric means of titers, and long dashed lines indicate the detection limit of HAI and MN assays. Data collected from 3 mice per group. * for $P<0.05$; **** for $P<0.0001$, calculated by two-way ANOVA followed by a Tukey's multiple comparison test.

References:

1. Kumar, S., et al., *MEGA X: Molecular Evolutionary Genetics Analysis across Computing Platforms.* Mol Biol Evol, 2018. **35**(6): p. 1547-1549.
